# Preclinical models of radiation-induced cardiac toxicity: Potential mechanisms and biomarkers

**DOI:** 10.3389/fonc.2022.920867

**Published:** 2022-10-12

**Authors:** Alexandra D. Dreyfuss, Anastasia Velalopoulou, Harris Avgousti, Brett I. Bell, Ioannis I. Verginadis

**Affiliations:** Department of Radiation Oncology, University of Pennsylvania, Philadelphia, PA, United States

**Keywords:** radiation therapy, radiation-induced heart disease (RIHD), cardiovascular toxicity, biomarkers, radiation-induced myocardial fibrosis, preclinical model, chemoradiotherapy, immunoradiotherapy

## Abstract

Radiation therapy (RT) is an important modality in cancer treatment with >50% of cancer patients undergoing RT for curative or palliative intent. In patients with breast, lung, and esophageal cancer, as well as mediastinal malignancies, incidental RT dose to heart or vascular structures has been linked to the development of Radiation-Induced Heart Disease (RIHD) which manifests as ischemic heart disease, cardiomyopathy, cardiac dysfunction, and heart failure. Despite the remarkable progress in the delivery of radiotherapy treatment, off-target cardiac toxicities are unavoidable. One of the best-studied pathological consequences of incidental exposure of the heart to RT is collagen deposition and fibrosis, leading to the development of radiation-induced myocardial fibrosis (RIMF). However, the pathogenesis of RIMF is still largely unknown. Moreover, there are no available clinical approaches to reverse RIMF once it occurs and it continues to impair the quality of life of long-term cancer survivors. Hence, there is an increasing need for more clinically relevant preclinical models to elucidate the molecular and cellular mechanisms involved in the development of RIMF. This review offers an insight into the existing preclinical models to study RIHD and the suggested mechanisms of RIMF, as well as available multi-modality treatments and outcomes. Moreover, we summarize the valuable detection methods of RIHD/RIMF, and the clinical use of sensitive radiographic and circulating biomarkers.

## Introduction

Current approaches to oncologic care rely heavily on multidisciplinary therapies delivered concurrently, sequentially, or in a response-adapted stepwise fashion. This strategy along with the use of novel therapies have contributed to significant increases in treatment response rates and long-term survival of cancer patients, leading to a population of cancer survivors of approximately 17 million in the United States alone, and 19.3 million new cancer diagnoses in 2020 worldwide (1, 2). However, as cancer patients are overcoming their diagnoses at increasingly convincing rates, they are also presenting clinicians with a new set of challenges involving the management of morbid and quality-of-life-limiting sequelae of their treatments. In particular, treatment-related cardiovascular mortality has become a major contributor to the global burden of cardiovascular disease, motivating a paradigm shift in the way we approach cardiovascular screening, risk stratification, diagnosis, treatment, and surveillance in cancer patients.

Cancer therapy-induced cardiovascular toxicity can manifest as a variety of clinical pathologies. The European Society of Cardiology guidelines have broadly categorized cardiotoxicity into several disease groups, including cardiomyopathy, myocarditis, pericardial disease, arrhythmias, myocardial ischemia, hypertension, vascular events, and valvular disease (3). The large spectrum of cardiovascular pathologies that have been observed is in part due to the large number of cardiotoxic agents increasingly used in oncologic care, including chemotherapies such as anthracyclines, targeted therapies such as tyrosine kinase inhibitors, immunotherapies such as immune checkpoint inhibitors, hormone therapies, and thoracic radiotherapy. This clinical heterogeneity is also dependent on multiple patient-specific factors, including age at therapy, sex, baseline cardiovascular risk, and overall health comorbidities (4). Moreover, throughout their disease course, many patients are increasingly receiving more than one cardiotoxic agent, further complicating the underlying disease mechanisms involved in causative pathophysiology.

Radiation therapy is one treatment modality with dose-dependent cardiotoxic potential (5–7). Radiation exploits a therapeutic index between normal tissue and tumor cells, causing differential cell death due to DNA damage and associated free radical formation, immune cell infiltration, and cytokine production. Despite vast improvements in radiation technologies and treatment delivery, such as Intensity-Modulated Radiotherapy (IMRT), Stereotactic Body Radiotherapy (SBRT) and proton therapy, incidental dose delivery to the heart is still a limiting factor in thoracic radiation therapy (8, 9). A recent large retrospective study reported that compared with non-irradiated patients, chest radiation therapy patients have a 2% higher absolute risk of cardiac morbidity and death at 5 years, and a 23% increased absolute risk at 20 years post-treatment (10). Furthermore, several large studies have demonstrated significant cardiovascular consequences of radiation therapy in breast cancer, lung cancer, and mediastinal lymphoma patient populations (10–13).

While recent research has provided significant insight, there remains a critical knowledge gap in pathophysiologic mechanisms of cardiovascular toxicity and optimal strategies for risk stratification, monitoring, and treating patients with cardiovascular dysfunction. This review describes the mechanisms by which thoracic radiation can cause therapy-induced myocardial fibrosis and discusses innovative preclinical models for studying such pathophysiology. We include an overview of the current landscape of radiographic and plasma biomarkers for therapy-induced toxicity, the beneficial effects and risks of multi-modality treatments and end with a discussion of future directions for the field.

## Preclinical models to study RIHD

Preclinical animal models of mice, rats, rabbits, dogs, pigs, and non-human primates have long been used in studies of RIHD (14–21). Favorable characteristics such as the low maintenance and housing costs, reduced gestational times and lifespan, as well as the convenience for genetic modifications and selections have rendered the rodents an excellent model to perform mechanistic studies.

Many manifestations of RIHD have been successfully modeled in rodents yielding a wealth of information regarding the molecular pathways that participate in the development of the radiation-induced cardiotoxicity (22–29). Several genetically engineered rodent models developed to be prone to cardiac pathologies, such as atherosclerosis, have been studied following radiation delivery (30–36). For instance, it has been demonstrated that inflammatory and thrombotic responses are accelerated post ionizing irradiation on atherosclerosis-prone ApoE(-/-) mice but not on wildtype C57BL/6J mice (32, 37, 38). Moreover, endothelial-specific knock-out mouse models of p53 and p21 have contributed to the delineation of the role of endothelial cells in cardiac damage post-radiation (39, 40). Therefore, it is of high importance the selection of the right rodent genetic background to study any aspect of RIHD (41). In addition, Cre-loxP technology has offered the opportunity to create more pronounced or rapidly developed phenotypes of cardiac toxicity (42–44). Cardiac radiotherapy has also benefitted from rodent models of partial heart irradiation which aid in the identification of molecular signaling pathways regulating the RIHD milieu (42, 45–47). However, rodent models pose some limitations such as their phylogenetic distance from humans, and their physiological responses to treatment schemes (48–50), thus there is an obvious need for larger animal models of RIHD.

Rabbits were introduced as another animal model to study the RIHD to avoid the limitations experienced by the rodent models. Due to several commonalities in cardiac physiology with humans (51–53), rabbits serve as an excellent model to study heart failure, ischemic heart disease, and electrophysiological phenomena caused by radiation and as an alternative choice to larger animals (51–55). Nevertheless, there are marked differences in the physical size of the rabbit heart, the heart rates, and body weight, and these facts ought to be taken into consideration especially in studies of arrhythmia as a side effect of radiotherapy (53, 56). The extensive use of rabbits in cardiac radiation research since 1968 (New Zealand white rabbits) (57) has resulted in a plethora of available transgenic rabbit models of cardiovascular disease and rabbit-specific antibodies, facilitating their extended use (53, 58–60).

On the contrary, significantly fewer canine models have been chosen in heart radiation studies (16, 61–64) despite the fact that canine models present many similarities on both the organ and cellular levels with humans. Moreover, it has been demonstrated that canine coronary circulation presents similarities to ischemic hearts of elderly people (65–67). This can be attributed to the fact that more strict regulations and regional approval procedures may apply to the use of dogs as well as to the very expensive housing and maintenance. Furthermore, another obvious obstacle is the human-canine bond developed by the society which provokes criticism for the canine studies (68–70).

Manifestations of RIHD are significantly common between pigs and non-human primates with humans (65–67) but these species have also been used by only a few groups as they carry significantly higher costs than other models (19–21, 71).

Although the choice of species, strain, and genotype needs extra attention, other parameters that need to be carefully chosen when designing studies to mimic a clinical scenario of RIHD include the animal age, size, and gender, as they can greatly influence the experimental findings (72, 73). Detailed reporting of these variables will allow for unbiased comparisons between studies.

## Radiation-induced myocardial fibrosis

Radiation-induced myocardial fibrosis (RIMF) is considered the final stage of RIHD (74–78) and is characterized by the excess collagen deposition in the damaged cardiac tissue (79–81); nevertheless, the molecular mechanisms remain unclear. RIMF increases the stiffness of the myocardium and decreases the systolic and diastolic functions, leading to the development of a myocardial electrical physiological disorder, arrhythmias, deficient heart function, or death (81). The etiology of the myocardial stiffness (23, 82, 83) lies in the increased presence of cytoplasmic actin stress fibers, on myofibroblasts producing collagen (84, 85) and on an activated inflammatory milieu (86, 87). Diverse mechanisms link RIMF with downstream pathologies, such as arrhythmia, cardiomyopathy, and myocardial ischemia. Fibrosis affecting the heart’s conduction system or cardiomyocyte conduction could interfere with the transmission of electrophysiological signals, causing arrhythmias like AV-nodal bradycardia or heart block (88). Similarly, microvascular damage may result in ischemia affecting these systems to cause these conduction abnormalities. Such microvascular damage is also mechanistically linked to cardiomyopathy following radiotherapy. The reduction of capillaries supplying cardiomyocytes results in hypoxia and death of myocardial tissue with progressive fibrosis. This reduces ejection fraction and increases left ventricular end-diastolic volume. One study of patients with left-sided breast cancer associated Tc-99m sestamibi perfusion deficits with wall motion abnormalities after radiation (89).

**Figure 1:**
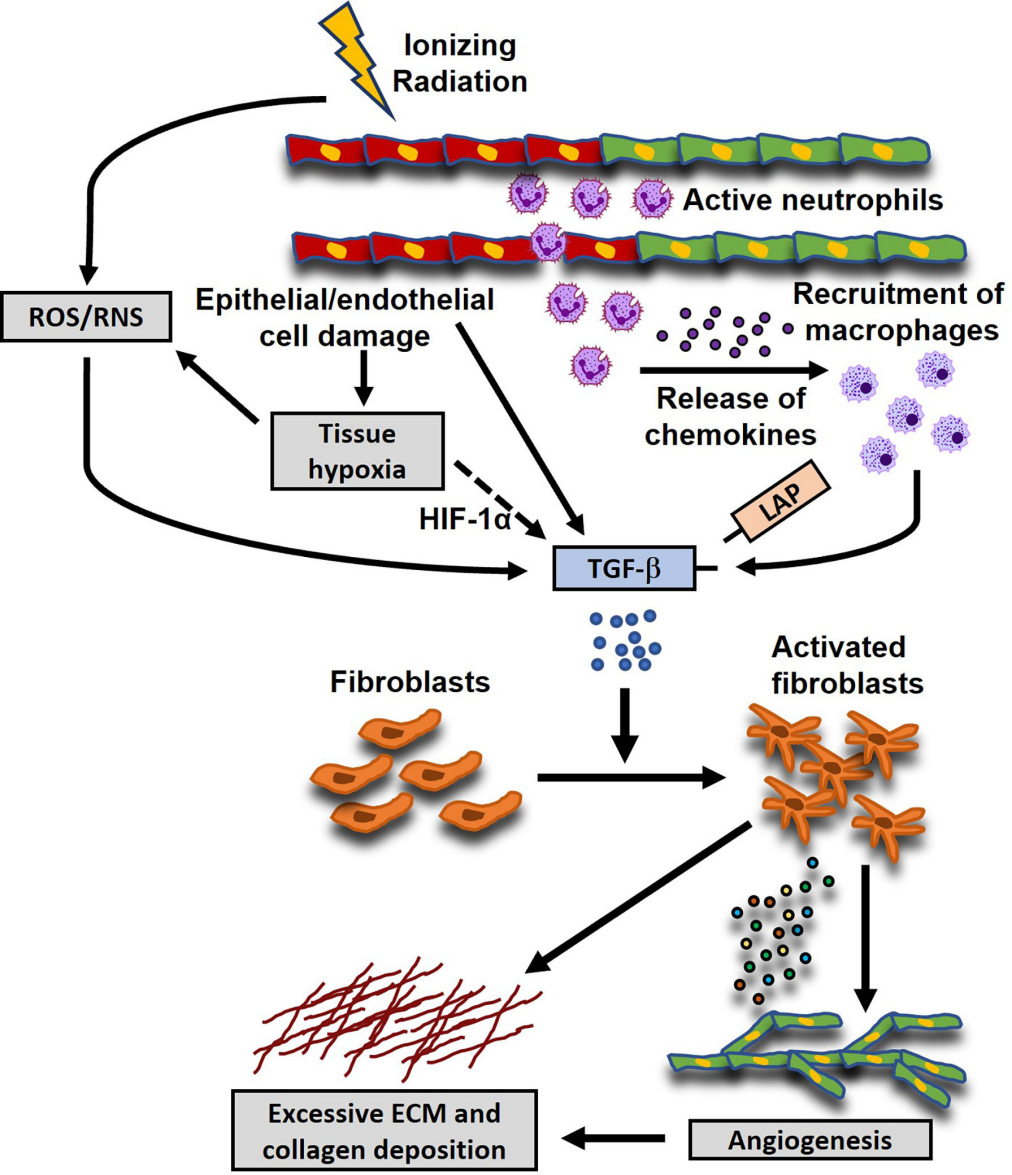
Proposed mechanisms of radiation-induced myocardial fibrosis (RIMF). Ionizing radiation damages epithelial and endothelial (shown here; red- and green-colored cells are damaged and healthy cells, respectively) cells which in turn induce a coordinated cellular response that also involves the secretion of pro-fibrotic cytokines, such as Transforming Growth Factor b (TGF-β). Moreover, the damaged endothelial cells secrete inflammatory chemokines to recruit neutrophils and macrophages to the injured sites, which in turn release pro-fibrotic cytokines like Platelet-Derived Growth Factor (PDGF), basic Fibroblast Growth Factor (FGF), TGF-β, etc., leading to a chronic inflammatory environment. Additionally, radiation causes perturbations of the cellular homeostasis by the excessive production of reactive oxygen and nitrogen species (ROS and RNS), which can also lead to the activation of TGF-β through the dissociation of the Latency Associated Peptide (LAP) from the active mature form of TGF-β. Another potential mechanism is that the damaged vasculature and the uncontrolled tissue remodeling can initiate the TGF-β signaling pathway through the ROS/RNS imbalance or the up-regulation of the expression of Hypoxia-Inducible Factor 1α (HIF-1α). The activated TGF-β signaling pathway leads to the differentiation and activation of the residual and migrated fibroblasts, characterized by the increased secretion of extracellular matrix (ECM) and collagen deposition.

The induction of oxidative stress (OS) and the triggering of an early pro-inflammatory environment by irradiation are considered major factors contributing to the initiation and development of RIMF. The inflammatory cascade is initiated by the vascular injury and endothelial dysfunction at approximately six hours post-irradiation (90, 91), which is followed by neutrophil adherence to the endothelium and migration to the damaged heart (92, 93). Various cytokines and growth factors, including Transforming Growth Factor β (TGF-β) (94–96), Tumor Necrosis Factor α (TNF-α) (97–99), Interleukin-1 (IL-1) (100, 101), Interleukin-11 (IL-11) (102–104), Connective Tissue Growth Factor (CTGF) (105), Platelet-derived Growth Factors (PDGFs) (106, 107), Vascular Endothelial Growth Factor (VEGF) and Fibroblast Growth Factor (FGF) (108, 109) flood the damaged heart a few hours after irradiation resulting in higher numbers of infiltrated neutrophils and lymphocytes (110). Additionally, monocytes differentiate into the M2 subtype of macrophages to start secreting TGF-β. In turn, TGF-β differentiates the resident fibroblasts and bone marrow progenitors (111) into myofibroblasts and this event is considered a hallmark of RIHD-related fibrosis (74, 112–114). Subsequently, activated myofibroblasts intensify the synthesis of extracellular matrix (ECM) which consists of a complex combination of collagens, glycoproteins, proteoglycans and matricellular proteins (109, 115). The excessive synthesis of ECM is accompanied by the increased expression of integrins, the tissue inhibitors of matrix metalloproteinases (MMP) (109, 112, 115). Also, the decreased cardiac contraction which has been associated with the development of arrhythmias and mortality can be triggered by the damage to the mechano-electric coupling of the cardiomyocytes caused by the excess ECM. Moreover, deprivation of oxygen and nutrients due to inflammation and fibrosis can further aggravate the adverse cardiac remodeling (116) and impair elasticity and distensibility, promoting ejection fraction and cardiac failure (110). Besides the inflammatory pathways, the proto-oncogenes c-Jun and c-Myc may contribute to the emergence of late fibrosis (74, 117). In addition to the inflammatory signaling, DNA damage response, chronic oxidative stress, chronic hypoxia, epigenetic regulation, and telomere extension have also been inculpated in the radiation-induced fibrosis and the related heart pathologies (74, 118). Another potential mechanism is that the radiation-induced DNA damage which cannot be efficiently repaired in the cardiac tissue (119) can eventually upregulate BAX and downregulate BCL2 in cardiomyocytes, contributing to apoptosis and subsequent development of fibrosis (120, 121) and the progression of the RIHD (39). The above-described mechanisms of radiation-induced myocardial fibrosis are depicted in **Figure 1**.

## Detection methods for RIHD/RIMF and biomarkers

**Figure 2:**
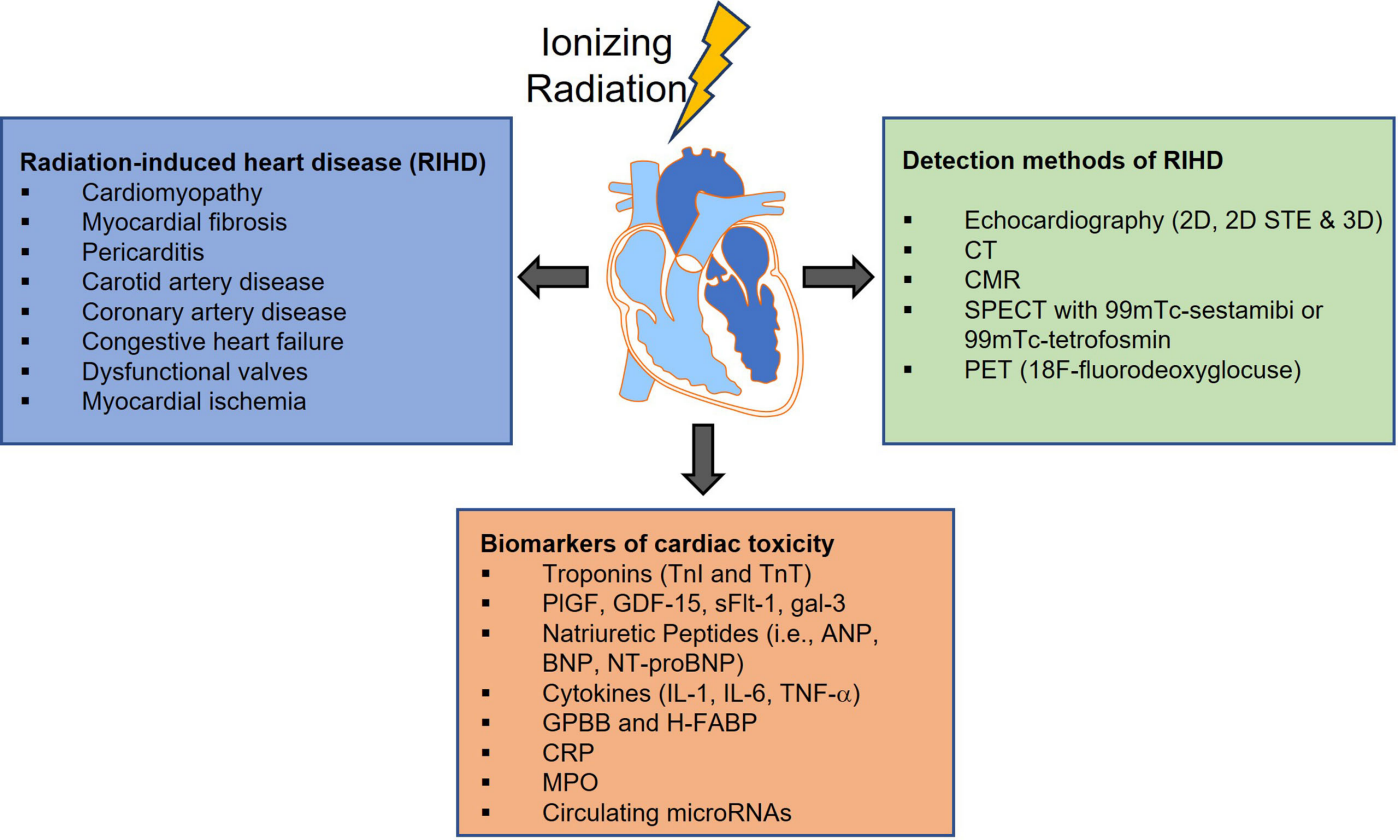
Radiation-induced cardiovascular toxicity can manifest as a variety of clinical pathologies. Although the implementation of technological improvements to the radiotherapy (RT) treatment, has led to more precise targeting and has allowed modest increases in RT dose to the tumor, incidental RT to the heart and development of RIHD is still unavoidable. Over the past decades, numerous methods have been developed for cancer therapy-related cardiovascular disease screening and diagnosis, and more particularly for diagnosing radiation-induced injury. However, cardiovascular disease is often diagnosed when damage is irreversible thus the identification and use of sensitive radiographic and circulating biomarkers to risk stratify or diagnose cardiotoxicity are urgently needed. 2D STE, Two-Dimensional Speckle Tracking Echocardiography; CT, Computed Tomography; CMR, Cardiac Magnetic Resonance imaging; SPECT, Single Photon Emission Computerized Echocardiography; PET, Positron Emission Tomography; PlGF, Placenta Growth Factor; GDF-15, Growth Differentiation Factor-15; sFlt-1, soluble fms-like tyrosine kinase receptor-1; gal-3, galectin-3; ANP, Atrial Natriuretic Peptide; BNP and NT-proBNP, Brain Natriuretic Peptide and its amino-terminal component; IL-1, interleukin-1; IL-6, interleukin-6, TNF-a, Tumor Necrosis Factor-alpha; GPBB, Glycogen Phosphorylase isoenzyme BB; H-FABP, Heart-type Fatty Acid-Binding Protein; CRP, C-Reactive Protein; MPO, Myeloperoxidase.

The current paradigm for cardiovascular care of oncology patients involves a number of steps once a cancer diagnosis is established. Standard practice involves baseline cardiovascular risk assessment based on medical and family history, age, obesity, smoking, and diabetes, cardiotoxicity monitoring during treatment, and long-term cardiovascular surveillance most commonly with serial echocardiography. However, cardiovascular disease is often diagnosed when damage is irreversible and is typically treated in a non-targeted fashion with routine cardioprotective medications such as beta-blockers or statins (122, 123). The identification and use of sensitive radiographic and circulating biomarkers to risk stratify or diagnose cardiotoxicity before tissue damage becomes irreversible is therefore an active area of research, with an overarching goal of informing more targeted and effective therapeutic intervention (**Figure 2**).

### Echocardiography

2D transthoracic echocardiography has been the most widely used imaging modality for screening baseline cardiovascular risk and for diagnosing cardiac injury, largely due to its widespread availability, non-invasiveness, cost-effectiveness, and validation in diagnosing non-oncologic cardiac disease. Clinicians have relied heavily on >10% decreases in left ventricular ejection fraction (LVEF) to diagnose myocardial dysfunction, and many clinical studies and national guidelines include LVEF changes by echocardiography as a primary or secondary outcome measure (124, 125). Still, 2D echocardiography-derived LVEF has its limitations, including operator variability and nontrivial false-negative rates. The use of more advanced techniques such as 3D echocardiography or ultrasound-based contrast agents has therefore been proposed as appealing alternatives due to their improved image acquisition.

Still, it is now widely accepted that measuring LVEF alone does not typically provide a sufficient assessment of a patient’s overall cardiac function. LVEF provides limited insight into underlying pathophysiology, for example, in discerning whether decreased LVEF is due to intrinsic myocardial injury or vascular damage-causing ischemia. Additionally, even though recovery of LVEF is possible if a diagnosis of the cardiac injury is made early enough, decreases in LVEF are often only detectable once tissue injury is irreparable. As such, LVEF measurements have been increasingly analyzed in conjunction with additional radiographic markers of cardiac mechanics, such as myocardial strain, torsion, and vascular stiffness (126). The global longitudinal strain has emerged as an attractive radiographic biomarker with higher sensitivity in diagnosing subclinical cardiotoxicity, largely due to the fact that changes in strain are usually seen prior to changes in LVEF (127, 128). The ongoing SUCCOUR study is prospectively investigating the initiation of cardioprotective medications following a decrease in global longitudinal strain compared to a decrease in LVEF (129). However, such biomarkers often require more advanced imaging techniques such as speckle-tracking echocardiography (130), which is not always routinely available, and further validation in large studies will be required prior to widespread implementation in the clinic.

### Cardiac magnetic resonance imaging

Cardiac magnetic resonance imaging (CMR) is a useful imaging modality that has the potential to address some of the limitations of echocardiography. For one, the high topographic resolution and reproducibility of CMR allows not only for sensitive diagnosis but also aids in phenotyping underlying pathology while sparing patients from repeated radiation exposure. Both T1 and T2 sequences that exploit differential water content and exchange of healthy and diseased cardiac tissue have been widely validated in non-oncologic populations. Changes in tissue structure are easily detectable with increases in T1 and T2, particularly for edema, inflammation, and fibrosis, all of which have been implicated in cancer therapy-related cardiac disease.

CMR also offers a mild but real improvement in measuring myocardial strain, and several studies have reported a decrease in myocardial strain measurements after anthracyclines (131–133). Following radiation therapy, several studies have reported changes in LVEF and left ventricular mass on CMR just 6 months after treatment (134, 135). Several “tagging” sequences, such as harmonic phase (HARP) or displacement encoding (DENSE), have also demonstrated utility in measuring myocardial movement during the cardiac cycle, which can detect subclinical cardiotoxicity early. Nevertheless, CMR also has its disadvantages, including high cost, long image-acquisition time, and limited availability. Until these challenges can be addressed, the use of CMR in cardio-oncologic populations will likely remain limited to those in whom echocardiographic assessments are of poor quality or inconclusive.

### Nuclear imaging

While echocardiography and CMR have been the most frequently used imaging modalities for cancer therapy-related cardiovascular disease screening and diagnosis, the role of nuclear imaging has received increasing interest, particularly for diagnosing radiation-induced injury. Recent research has demonstrated that radiation-induced cardiovascular damage is driven at least in large part by inflammatory cell activation and plaque formation along with direct vascular cell damage. The most basic consequence of these events is decreased coronary circulation and myocardial perfusion deficits. Single-photon emission computerized echocardiography (SPECT) with 99mTc-sestamibi or 99mTc-tetrofosmin is a widely recognized imaging modality for the detection of myocardial perfusion deficits secondary to myocardial fibrosis and/or endothelial cell dysfunction. To date, multiple studies have investigated the use of SPECT imaging in thoracic radiation patients and have found convincing associations between perfusion deficits and volume and location of the irradiated field, supporting a volume and dose-dependent relationship between radiation and cardiovascular damage (136–140). However, SPECT imaging is limited by its relatively low spatial resolution and can therefore fail to reveal microvascular dysfunction or perfusion deficits in less easily detectable locations. Additionally, SPECT involves radiation exposure and can be relatively time-consuming for patients due to the long half-life of SPECT isotopes.

Positron emission tomography (PET) imaging for cardiac disease is another imaging modality that has been increasingly implemented, with the potential to identify a variety of distinct cardiac pathologies depending on the radiotracer used (141, 142). For example, 18F-fluorodeoxyglucose (18F-FDG) is a well-known marker of inflammation in the body, and in the case of cardiac PET, 18F-FDG can identify inflammation in the cardiac vasculature which often precedes atherosclerotic plaque progression and rupture (143, 144). Patient preparation for cardiac PET does involve a fasting period of 12-18 hours with or without a diet low in carbohydrates for 12-24 hours to suppress basal myocardial glucose update. Nevertheless, a major advantage of FDG-PET imaging is its ability to detect early metabolic changes in the myocardium that occur prior to decreases in LVEF.

It is also worth mentioning a number of novel molecular tracers that have become more widely available in clinical care in recent years. For example, tracers such as Rubidium-82 and [13N]Ammonia are particularly useful for measuring myocardial blood flow in the microvasculature, offering higher resolution than SPECT (145). Additionally, ^99m^Tc-labeled annexin V, a plasma protein that can detect cellular apoptosis in unstable plaques, has already been used to reveal dose-dependent cell death prior to changes in echocardiography (146). Investigation in large cohorts is warranted and will be necessary to establish the role of these radiotracers in diagnosing and monitoring subclinical cardiotoxicity in cancer patients.

### Plasma biomarkers

Identifying and monitoring cardiovascular toxicity during and after treatment with blood-based biomarkers is an attractive clinical approach that can complement radiographic assessments. Circulating biomarkers are typically reproducible and easily obtainable measures that do not rely on operator skill. Additionally, circulating biomarkers have the added benefit of reflecting underlying disease pathophysiology. All biomarkers that mentioned in this section and are related to radiation-induced heart disease as well as the putative biomarkers of cardiac toxicity associated with cancer treatment are summarized in **Tables 1** and **2**, respectively.

**Table 1:** Summary of biomarkers studied in radiation-induced heart disease in humans.

Reference	Tumor Type	Treatment	Mean Heart RT Dose	Detection Time Post-Treatment	Tn	BNP	NT pro-BNP	CKMB	Gal-3	MPO	PIGF	GDF-15	c-miRNA	Notes
(125)	Lung Cancer	Radiotherapy	8.4 Gy	Median of 20 days post-RT	n.d.	–	n.d.	–	–	↑	↑	–	–	Changes in biomarkers were not associated with echocardiographic evidence of toxicity.
	Lymphoma		6.8 Gy		n.d.	–	n.d.	–	–	↑	↑	–	–	
	Breast Cancer		1.3 Gy		n.d.	–	n.d.	–	–	n.d.	n.d.	–	–	
(147)	Lung Cancer	Radiotherapy or Chemoradiation	13.7 Gy	Pre-treatment	–	–	–	–	–	–	–	–	↑	High pre-treatment miRNA signature predicts toxicity.
(148)	Breast Cancer	Radiotherapy	2.39 Gy	1, 6, 12 months post-RT	–	↑	–	–	–	–	–	–	–	BNP normalized to pre-treatment, but not raw BNP, correlates with toxicity.
(149)	Breast Cancer	Radiotherapy	9.4 Gy	First treatment, mid-treatment, last treatment, and 6 months post-RT	n.d.	–	n.d.	–	n.d.	–	–	n.d.	–	
(150)	Primary Thoracic Malignancies	Radiotherapy or Chemoradiation	26.5 Gy	Last day of RT, 1-2 months post-RT	n.d.	↑	–	–	–	–	–	–	–	
(151)	Breast Cancer	Radiotherapy	2.5 Gy	5-22 months post-RT	n.d.	–	↑	–	–	–	–	–	–	
(152)	Lung and Breast Cancer	Radiotherapy	–	Weekly for 6 weeks on-treatment	↑	↑	–	–	–	–	–	–	–	
(153)	Thoracic malignancies	Radiotherapy or Chemoradiation	13.4 Gy	After 2 weeks of treatment and end of treatment.	n.d.	–	n.d.	n.d.	–	–	–	–	–	
(154)	Breast Cancer	Chemoradiation	–	Median of 6.5 years post-treatment	–	↑	–	–	–	–	–	–	–	

RT, Radiotherapy; n.d., no difference; -, not assessed; Tn, Troponin; BNP, B-type natriuretic peptide; NT pro-BNP, N-terminal pro-BNP; CKMB, Creatine Kinase-MB; Gal-3, galectin-3; MPO, myeloperoxidase. PlGF, Placental growth factor. GDF-15, Growth/differentiation factor-15. ↑, increased levels.

**Table 2:** Summary of putative markers of cardiac toxicity associated with cancer treatment.

Reference	Tumor Type	Treatment	Detection Time Post-Treatment	Tn	BNP	NT pro-BNP	CKMB	Gal-3	MPO	PIGF	GDF-15	c-miRNA	CRP	s-Flt1	H-FABP	GPBB	Notes
(155)	Breast Cancer	Chemotherapy	3 months	↑	–	n.d.	–	n.d.	↑	↑	↑	–	↑	↑	–	–	Only TnI and MPO correlated with cardiotoxicity risk.
(156)	Breast Cancer	Trastuzumab	3 month intervals	–	–	–	–	–	–	–	–	–	↑	–	–	–	Maximum CRP correlates with toxicity
(157)	Lymphoma	Chemotherapy	24 hours after 1st dose (H-FABP) and after last dose (BNP)	–	↑	–	–	–	–	–	–	–	–	–	↑	–	Early H-FABP correlated with late BNP and reduced ejection fraction.
(163)	Leukemia/ Lymphoma	Chemotherapy		n.d.	–	–	n.d.	–	–	–	–	–	–	–	n.d.	↑	GPBB increase correlated with LV diastolic dysfunction.

CRP, C-reactive protein; sFlt-1, soluble fms-like tyrosine kinase receptor-1; GPBB. -, not assessed; ↑, increased levels.

Cardiac troponins and natriuretic peptides have been at the center of cardiovascular disease diagnosis both in oncologic and non-oncologic patient populations. Similar to well-known markers of myocardial stress and heart failure, increases in troponin and B-type natriuretic peptide (BNP) have been used to signal cardiac injury at various stages in a patient’s cancer treatment, including in baseline risk assessment, monitoring during therapy, and surveillance after therapy (158, 159). To date, multiple studies have evaluated increases in troponin and BNP following cardiotoxic therapy administration with some promising but mixed results (148–155, 160, 161). A recent meta-analysis of 61 trials including 5691 cancer patients reported that anticancer therapy was associated with increases in the levels of troponin was in turn associated with a higher risk for cardiac dysfunction (160). Nevertheless, mixed study results have highlighted the need for more sensitive and specific biochemical markers of cardiovascular damage (162).

Several novel circulating biomarkers that have been proposed include markers of inflammation, oxidative stress, fibrosis, angiogenesis, and vascular remodeling, including cytokines, interleukins, myeloperoxidase, C-reactive protein, galectin-3, placental growth factor, and growth differentiation factor-15 (**Figure 2**) (157, 163). For example, one study involving 54 women with human epidermal growth factor receptor 2 (HER2)-positive early-stage breast cancer treated with trastuzumab showed that peak levels of high-sensitivity C-reactive protein (hs-CRP) were detected after a median of 78 days before decreases in LVEF were observed (156). Moreover, biomarkers analysis in a multicenter study of 78 patients with breast cancer demonstrated early increases in myeloperoxidase and Troponin I (TnI) levels following treatment with doxorubicin and trastuzumab (155). Additionally, microRNAs which mediate cardiac hypertrophy and fibrosis, and genome-wide association studies represent relatively untapped resources in the field of cardio-oncology (147, 164–167). Their role in diagnosing cardiovascular dysfunction remains to be determined in this patient population.

### Preclinical endpoints for RIHD

Preclinical studies benefit from many of the same techniques used to assess cardiovascular dysfunction in patients, but additionally see more frequent use of the gold standard for detecting RIMF: histologic analysis of cardiac tissue. Colorimetric stains, such as Masson’s trichrome (47) and picrosirius red (168), have been used to detect and quantify extracellular matrix components in preclinical models of RIHD. Similarly, more advanced microscopy techniques like second-harmonic generation (169) or atomic force microscopy (170) have been used to assess fibrillar collagen structure and tissue stiffness in other cardiovascular diseases. These techniques may provide insight into the supramolecular assembly of collagen and how it limits contractility in RIHD. The spatial dimension of cardiac histology allows for the assessment of cell-cell interactions, immune cell infiltration, and patterns of gene expression. Especially with the advent of novel spatial transcriptomic (171), tissue-based analysis can provide additional insight into the genetic programs induced in and around regions of RIMF.

Meanwhile, transthoracic echocardiography (TTE), CMR, or nuclear medicine studies allows for functional analysis in mouse models of RIHD. Small animal TTE platforms may be utilized to assess LVEF, ventricular wall motion abnormalities, valvular disease, and other pathologies in rodent RIHD studies (172), while clinical ultrasound machines are sufficient for larger animal studies. Cardiac MRI using high-resolution small animal MRI scanners offer similar endpoints as TTE for use in studies of RIHD (173). Similarly, Tc-99m-Sestamibi SPECT has been utilized to detect perfusion deficits in studies of RIHD (47) and myocardial infarction (174). Furthermore, animal models excel in biomarker detection because serial blood sampling for analysis of serum proteins may be correlated with imaging and/or histologic findings in the same animal.

## Synergistic cardiotoxicities with chemoradiation or radioimmunotherapy

Multiple studies have shown the clinical and therapeutic implications of combining radiotherapy with established chemotherapy and/or immunotherapy in the treatment of solid tumors (175–177). However, these potent and intense anti-tumor combined therapies have been associated with considerable tissue toxicities, most notably cardiotoxicities, which may adversely affect the quality associated with the improved outcome. These toxicities have been well characterized and remain a focus area for improving the therapeutic index of thoracic tumor treatment. Therefore, oncologists and cardiologists are called to design a multimodality treatment approach by integrating the latest clinical findings in cardiotoxicity, to achieve the best overall care for cancer patients undergoing chemoradiotherapy and/or immunoradiotherapy.

### Chemotherapy and radiation

The use of concurrent chemotherapy and radiotherapy (cCRT) against a variety of solid tumors, such as esophageal, breast, and non-small cell lung cancer (NSCLC) has been a promising strategy for the synergistic enhancement of local and distal tumor control, which in many cases has been translated into survival benefits (178–182). Moreover, numerous preclinical studies have also described that the combination of chemotherapeutic agents with radiotherapy could be a promising strategy for synergistic enhancement of treatment efficacy (183, 184). However, this multi-modality treatment has also been shown to increase risks for adverse cardiac events such as ischemic heart disease, myocardial infarction, heart failure, and cardiac dysfunction (185–187). These side effects can vary among individuals and are often correlated with the cumulative dose of the treatment.

In a retrospective analysis of a large cohort of older patients with NSCLC, patients who received radiotherapy or chemoradiotherapy treatment in the left lung had a significantly increased risk of ischemic heart disease compared to those who received either radiation-only (HR = 1.18, 95% CI 1.05–1.34) or chemoradiation (HR = 1.29, 95% CI 1.09–1.52) treatment in the right lung (187). Similar findings have been reported in other studies showing that radiotherapy to the left chest has been linked to a higher risk of cardiac toxicity and subsequent risk of developing chronic cardiac disorders compared to the right chest radiotherapy (188–190). Additionally, in another retrospective analysis of NSCLC patients, there was an increased risk of major adverse cardiac events (cardiac death, unstable angina, myocardial infarction, heart failure hospitalization, or coronary bypass grafting) in patients receiving thoracic chemoradiotherapy (adjusted hazard ratio: 1.05/Gy) (191). Chemoradiotherapy is also an effective treatment in the management of superficial esophageal cancer. In a retrospective analysis of 80 patients with submucosal invasive non-metastatic esophageal cancer treated with 5-fluorouracil or cisplatin and 60 Gy in 30 fractions, 13 patients developed severe cardiac events and the 5-year cumulative cardiac event occurrence rate was 16.3% (192). In the same study, they also showed that the level of the heart’s exposure to radiation was a major prognostic factor for these occurrences. Patients exposed to radiation with more than 280 mL of V5000 cGy had a 16.8 times more likely chance of developing these cardiac events than those who received a smaller volume (192). In a prospective study of patients with non-metastatic esophageal cancer, the authors showed that 30% of the patients developed myocardial fibrosis and/or reversible ischemia as early as 3 months following neoadjuvant chemoradiation (193). Furthermore, several large-scale studies have demonstrated similar findings that chemoradiotherapy increases the risk of cardiotoxicity in lymphoma (194, 195) and esophageal cancer treatment (196) as compared to radiotherapy alone. The design of comprehensive treatment strategies will allow patients to receive potentially curative benefits from multi-modality treatments and minimize the cardiac-related toxicities.

### Immunotherapy and radiation

The discovery of the immune-checkpoint inhibitors [(ipilimumab, an anti-cytotoxic T lymphocyte-associated protein 4 (CTLA-4) antibody, programmed death 1 (PD-1) and programmed death-ligand 1 (PD-L1)], has placed immunotherapy to the front-line of cancer treatment, especially for advanced NSCLC (197, 198). Furthermore, in some types of cancer, immunotherapy has proven more effective (as a monotherapy) than the standard care treatment. This success also encouraged researchers to combine immunotherapy with other conventional therapies, i.e., radiotherapy, to improve the effectiveness. As in chemoradiotherapy, the combination of immunotherapy with radiotherapy has shown synergistic effects in both local and systemic tumor control (199–201), possibly due to the synergistic immune activation. For instance, in a phase III randomized-controlled, double-blinded trial with stage III non-small-cell lung cancer patients undergoing chemoradiotherapy, the addition of durvalumab (anti-PDL1 antibody) showed a significant increase in progression-free survival (198). Interestingly, an abscopal effect has also been reported post combined radiotherapy with immune checkpoint inhibitors (202, 203). Moreover, multiple preclinical studies have also demonstrated synergistic effects of combined immunotherapy with radiotherapy on treatment efficacy (202, 204–206). Although these studies support the beneficial effects of immunoradiotherapy in tumor control mainly through increased immune-mediated antitumor activity, they also resulted in increased rates of cumulative cardiotoxicity (207). In a preclinical study by Du et al., the authors reported radiation-induced cardiac dysfunction and acute fibrosis which was exacerbated by the concurrent treatment with PD-1 blockade (208). In another *in vivo* study, the combination of thoracic irradiation with anti-PD-1 antibody significantly decreased the survival of the C57Bl/6 mice compared to radiation alone and that was positively correlated with the T cell infiltration into the lung and heart tissues (209).

Although the clinical use of immune checkpoint inhibitors in cancer treatment is exponentially increased in the last decade, the pathology and treatment of the immune-related toxicities are complex and still not well known. Therefore, more screening procedures and extensive research on the pathophysiology of cardiotoxicity are required. Additionally, the optimization of the radiation treatment planning (dose, timing, etc.) and the identification of sensitive biomarkers of cardiotoxicity will give further access to the promising treatment strategy of immunoradiotherapy.

### Targeted therapies and radiation

Similar to immunotherapy, targeted therapies are now commonly used in the comprehensive care of cancer patients who also often receive chemotherapy or radiation. However, there is little data regarding the combined impact of various targeted therapies and radiotherapy on cardiotoxicity. A few studies have assessed the combination of radiotherapy and Trastuzumab, a monoclonal antibody used in the treatment of HER2 receptor positive breast cancer. Three studies found no increase in acute cardiotoxicity in breast cancer patients treated with chemoradiotherapy and trastuzumab compared to chemoradiation alone, suggesting this combination therapy is clinically feasible (210–212). Yet, these studies are potentially confounded by the cardiotoxicities associated with anthracycline-containing chemotherapy regimens which limits our understanding of the toxicities induced by the combination of radiotherapy and trastuzumab. A clinical trial which assessed left-sided radiotherapy with concurrent trastuzumab compared to radiotherapy alone found a non-significant increase in the incidence of LVEF dysfunction (7.8% vs 4.1%, respectively), though treatment was generally well-tolerated. They also noted that the sequencing of RT and trastuzumab was an independent risk factor for the development of cardiotoxicity (213). This suggests that further study is warranted to understand the adverse effects of this combination. More generally, potential additive or synergistic toxicities of other targeted therapies used in combination with radiotherapy (and the associated incidental heart irradiation) should be investigated in the context of RIHD due to the limited clinical datasets available. For example, tyrosine kinase inhibitors, such as Osimertinib which is used in lung cancer, or anti-angiogenic agents, like bevacizumab, have been found to increase the risk of adverse events in patients receiving thoracic radiotherapy (214, 215). However, cardiotoxicity has not been specifically investigated in these settings. This is an area where the use of well-controlled preclinical models may prove useful, given how many potential combinations exist between targeted therapies and thoracic radiotherapy.

## Conclusions

Despite the advances in the delivery of thoracic radiation therapy, radiation-induced heart disease constitutes a growing clinical issue that severely compromises the quality of life of cancer survivors treated with thoracic RT alone or in combination with genotoxic chemotherapy or/and immunotherapy. Currently, as the multi-modality treatments have been considered successful approaches for cancer therapy, we need to reconsider the potential synergistic cardiac-related adverse effects. Although there is considerable progress in delineating the molecular and pathophysiological mechanisms behind the RIHD, the underlying development of RIHD still remains not thoroughly understood, therefore, new strategies need to be developed that can ameliorate or even reverse the course of RIHD. Therefore, there is an increasing need for more clinically relevant preclinical models to identify biomarkers and targetable mediators of RIHD which will lead to the development of prognostic screening techniques and new treatment options.

## Methods

Methods: A literature review of publications describing the preclinical models of RIHD/RIMF, the radiographic and circulating biomarkers, synergistic heart toxicities seen in radiotherapy combined with chemo/immunotherapy was performed. Specific databases utilized included PubMed and clinicaltrials.gov. Additionally, specific keywords have been used such as “Radiation-Induced heart disease AND rodents”, “Radiation-Induced heart disease AND rabbits”, “Radiation-Induced heart disease AND canine”, “Radiation-Induced heart disease AND pigs”, “Radiation-Induced heart disease AND non-human primates”, “Radiation-Induced myocardial fibrosis AND mechanisms AND rodents”, “Radiation-Induced myocardial fibrosis AND factors”, “Radiation-Induced myocardial fibrosis AND biomarkers”, “Radiation-Induced myocardial fibrosis AND radiographic”, “Radiation-Induced heart disease AND preclinical endpoints”, “cardiac toxicity AND radiation AND chemotherapy”, “cardiac toxicity AND radiation AND immunotherapy”, and other closely related terms.

## Author contributions

AD, AV, HA, BB, and IV conceptualized, wrote, and reviewed the manuscript. All authors contributed to the article and approved the submitted version.

## Funding

This work was partially supported by the National Center for Advancing Translational Sciences of the National Institutes of Health under Award Number UL1TR001878 and the Institute for Translational Medicine and Therapeutics’ (ITMAT) Transdisciplinary Program in Translational Medicine and Therapeutics to IV. The content is solely the responsibility of the authors and does not necessarily represent the official views of the NIH.

## Conflict of interest

The authors declare that the research was conducted in the absence of any commercial or financial relationships that could be construed as a potential conflict of interest.

## Publisher’s note

All claims expressed in this article are solely those of the authors and do not necessarily represent those of their affiliated organizations, or those of the publisher, the editors and the reviewers. Any product that may be evaluated in this article, or claim that may be made by its manufacturer, is not guaranteed or endorsed by the publisher.
